# Machine Learning Model for Nd_2_Fe_14_B-Based Permanent Magnets

**DOI:** 10.3390/ma19122643

**Published:** 2026-06-18

**Authors:** Manuel Enns, Wolfgang Körner, Daniel F. Urban, Christian Elsässer

**Affiliations:** 1 Fraunhofer Institute for Mechanics of Materials IWM, Wöhlerstr. 11, 79108 Freiburg, Germany; wolfgang.koerner@iwm.fraunhofer.de (W.K.); christian.elsaesser@iwm.fraunhofer.de (C.E.); 2 Freiburg Materials Research Center, University of Freiburg, Stefan-Meier-Str. 21, 79104 Freiburg, Germany

**Keywords:** permanent magnets, machine learning, density functional theory

## Abstract

We demonstrate an efficient machine learning (ML) model for the prediction of magnetic property changes in ${\mathrm{Nd}}_{2}$ ${\mathrm{Fe}}_{14}$B-based permanent magnets given a large range of different impurity elements. We show that relatively simple models can be sufficient to capture complex changes in the saturation magnetization $M_{s}$ and the magnetocrystalline anisotropy constant $K_{1}$. As the necessity for recycling the raw material of permanent magnets increases, the variety of impure chemicals and their concentrations increase as well. Some chemical elements with antiferromagnetic or complex magnetic ground states like Cr, Mn and Sm pose difficulties in the training of an ML model that can be effectively mitigated by feature engineering. This enables us to create a single model capable of describing more than twenty substitutional elements in a wide range of concentrations.

## 1. Introduction

Machine Learning (ML) has been demonstrated to be effective in describing magnetic properties for hard magnetic materials from theoretical [1,2] and experimental [3,4,5] training datasets or combinations of both [6,7]. The use of ML eliminates the need to computationally or even experimentally screen through every possible chemical composition of a hard magnetic phase. While in the past ML has often been used to search for new magnetic materials or to identify general trends in the magnetic properties, we focus on the effect of impurity elements on intrinsic hard-magnetic properties of intermetallic Nd-Fe-B phases.

The development of ML models for hard-magnetic materials focuses on constructing appropriate descriptors. Pham et al. have used the orbital field matrix [8,9], which is derived from the valence configurations of local neighborhoods in a crystal. Dam et al. have used kernel ridge regression to analyze the importance of different atomic properties of the transition-metal atoms and rare-earth atoms as well as the structural information of the system for an inference of Curie temperatures in Nd-Fe-B based magnets [10]. A higher complexity in the descriptor often comes at the cost of less flexibility and lower generalizability of the model. It should be assessed for each task whether a simpler description of the system at hand may work as well without sacrificing quality.

Our topic of interest is the hard magnetism of ${\mathrm{Nd}}_{2}$ ${\mathrm{Fe}}_{14}$B-based materials. These magnets dominate the market of permanent magnets [11], and the demand to efficiently recycle them is increasing [12]. During the process of recycling hard-magnetic materials, different elements can be incorporated into the new material that change the magnetic properties compared to a magnet made of pure raw materials. Sources of contamination are, for example, incompletely separated magnets with different compositions or residuals from surface coatings. Therefore, the influence of different impurities on the magnetic properties needs to be explored in order to determine quality tolerances for certain chemical elements in recycled products.

Our system of interest, crystalline ${\mathrm{Nd}}_{2}$ ${\mathrm{Fe}}_{14}$B, has 68 atoms in the tetragonal unit cell. Taking impurity elements into account, there are trillions of possible phases. Given a relatively small dataset of intrinsic hard-magnetic properties calculated with an efficient method of density functional theory (DFT) for a few thousand compositions, we investigate the capabilities of ML models to capture the particular trends in the magnetic properties of these rare-earth–transition-metal compounds. We extend the work of Möller et al. [1] for the rare-earth lean ${\mathrm{ThMn}}_{12}$-type phases to the crystallographically more complex ${\mathrm{Nd}}_{2}$ ${\mathrm{Fe}}_{14}$B phases. For this, we develop machine learning models that can handle multiple rare-earth, transition-metal and light main-group elements at the same time. This proof of concept should be readily extendable to larger sets of data, calculated or measured more precisely, as we expect similar trends and challenges to show up. Complementary to various ML models of increasing complexity being currently developed for various material properties, we want to illustrate how a rather simple model is sufficient for this task after incorporating physically motivated adjustments. We have built ML models for intrinsic magnetic properties, which can handle a wide range of different elemental compositions and concentrations. In the following, we describe and discuss their advantages and challenges.

## 2. Materials and Methods

### 2.1. Data Generation

The training and test data for the machine learning (ML) model are generated by calculating the magnetocrystalline anisotropy constant *K*_1_, the saturation magnetization *M*_s_ and the relative phase formation energy Δ*E*_f_ using a computational high-throughput-screening (HTS) workflow as described in Refs. [13,14,15]. High values for both $M_{s}$ and $K_{1}$ are desirable for a good permanent magnet, because it means that the maximum achievable magnetic field using the magnet is high and that it requires a strong external magnetic field to turn its magnetization direction.

**Figure 1:**
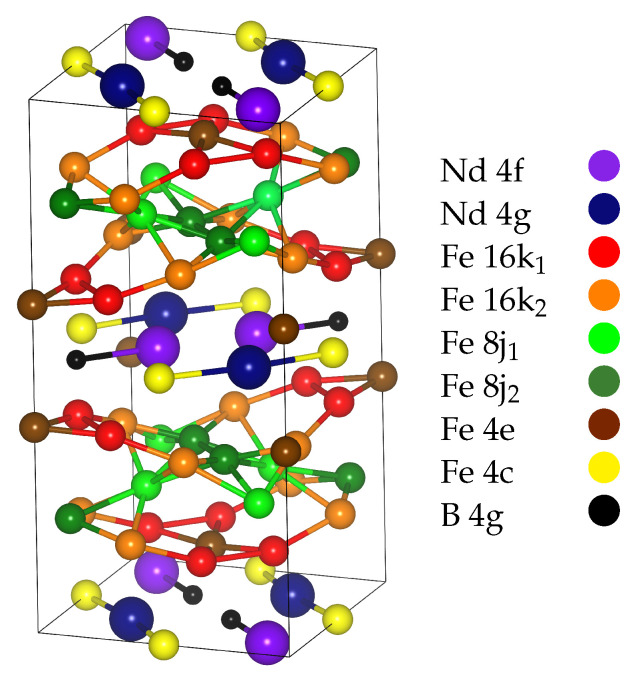
Crystal structure of ${\mathrm{Nd}}_{2}$ ${\mathrm{Fe}}_{14}$B with all nine Wyckoff positions indicated by different colors. The c-axis is stretched by a factor of 1.5 for better visualization of the individual atoms. (cf., e.g., Figure 1 in Ref. [21]).

The starting point for the calculations is the crystal structure of the Nd_2_Fe_14_B type [16,17,18], which has a tetragonal symmetry (space group P42/mnm, a=0.88 nm, c=1.22 nm) and nine distinct Wyckoff positions (see Figure 1). In the HTS, the Nd, Fe and B atoms are substituted with impurity elements by fully exchanging all atoms in one or more types of Wyckoff positions. As full substitutions of an element are unrealistic in a recycling process, we focus on lower impurity concentrations. This means that Fe is substituted by the elements *M* = Al, Ti, V, Cr, Mn, Co, Ni, Cu, Zn, Ga, Nb, and Mo for up to 23.5% (full substitution of the Wyckoff position with the highest multiplicity, i.e., ${16k}_{1}$ or ${16k}_{2}$, cf. Figure 1). These crystals are generated for Nd_2_${\mathrm{Fe}}_{14-x}$$M_{x}X$ with the light interstitial elements X= B, C, N, O and NdRE${\mathrm{Fe}}_{14-x}$$M_{x}$B with the rare earth elements RE= La, Ce, Pr, Sm, Gd, Tb, Dy, Ho. The chosen substitutional elements are typical impurities due to insufficiently separated scrap magnets, residual coating material, or further contaminations during the recycling process.

Note that not all of these elements would perfectly substitute the atoms in the crystal structure of the 2-14-1 phase under real conditions but segregate to grain boundaries or form nonmagnetic or soft-magnetic secondary-phase precipitates [19,20]. This work focuses on the proof of concept that illustrates the ML ability to describe and infer intrinsic magnetic properties over a large configurational space.

The calculations were done with the *tight-binding linear-muffin-tin-orbital atomic-sphere-approximation* method (TB-LMTO-ASA) [21,22,23,24,25]. For all the studied crystal compositions, the unit cells contain 68 atoms and have the same lattice parameters, as the structural distortions by compositional variations are negligible [26]. For a more detailed discussion on the influence that the fixed cell size has on the magnetic properties, we refer to previous studies on similar materials [15,27]. To summarize briefly, the change in $M_{s}$ is small (within 0.05 T), but $K_{1}$ is reported to be underestimated by several MJ/$m^{3}$. Nevertheless, the relative magnitude of $K_{1}$ was robust, and therefore our model, which aims at a relative comparison of the magnetic properties of compositions with different impurity concentrations, should not be affected drastically.

Δ*E*_f_ is calculated with respect to the most stable crystal phases of the individual impurity elements and is therefore only a crude estimate of the thermodynamical stability. Given an impurity concentration, there are several possibilities to place the elements on different Wyckoff positions, and Δ*E*_f_ is used to indicate the most stable configuration. After filtering and removing configurations with Δ*E*_f_ > 0.3 eV/atom or *M*_s_ < 0.25 T, our dataset consists of 2718 configurations. Due to different combinatorial possibilities for RE, *M* and *X* elements and a different amount of filtered configurations, there is a varying number of compositions containing a certain element. Nd and Fe is in every configuration, B is in 2363 configurations, all RE and almost all *M* elements (besides Nb and Ga with 51 and 49 configurations, respectively) are in 230 to 300 configurations, and C, N and O are in 137, 110 and 108 configurations, respectively.

### 2.2. Descriptor

The compositions in our database are described with the Wyckoff descriptor according to the approach outlined in Ref. [1]. In the latter work, it was used for hard-magnetic phases of the ${\mathrm{ThMn}}_{12}$-type with a single rare earth element. Each feature $n_{i}^{j}$ in the descriptor is an occupation number of a Wyckoff position i=1,…,P filled with an element j=1,…,N.

(1) $$x=\langle n_{1}^{\left(1\right)},\ldots ,n_{P}^{\left(1\right)},n_{1}^{\left(2\right)},\ldots ,n_{P}^{\left(2\right)},\ldots ,n_{1}^{\left(N\right)},\ldots ,n_{P}^{\left(N\right)}\rangle ,$$

If a Wyckoff position is never occupied by an element, that feature is omitted from the descriptor. For the purpose of comparing the magnetic properties of compounds with different impurity concentrations, it is useful to allow for continuous occupation numbers. Although our database only includes feature vectors with integer numbers of atoms, therefore containing compositions with impurity concentrations c with the stepsize of at least $\frac{1}{68}$, the feature vector naturally supports any real number for $n_{i}^{j}$. This enables a meaningful estimation of the magnetic properties for, e.g., $n_{4c}^{\mathrm{Co}}=0.5$, which is not possible for descriptors that require integer numbers of atoms.

### 2.3. Machine Learning

We use the Support Vector Regression (SVR) method [28,29,30] to build three individual ML models for the estimation of *K*_1_, *M*_s_ and Δ*E*_f_. The output values of the models $y^{m}$ are the estimations for these three properties and are calculated for an input feature vector **x** using the equation

(2) $$y^{m}\left(x\right)=\sum_{i}w_{i}k\left(x_{i},x\right)+b,$$

where the $w_{i}$ are the weights for each support vector $x_{i}$, $k(x_{i},x)$ is the kernel function, and *b* is the constant bias. The choice of kernel function in SVR, such as linear, polynomial, or radial basis function, allows for the effective modeling of non-linear relationships. We use the polynomial kernel

(3) $$k\left(x_{i},x\right)={\left(\gamma (x_{i}\cdot x)+1\right)}^{d}$$

with degree d=2. The kernel compares the similarity of **x** to the support vectors $x_{i}$ in the kernel space. Training the models minimizes this loss function L with

(4) $$\begin{array}{cc} L & =\frac{1}{2}{∥w∥}^{2}+C\sum_{j}\xi_{j} \end{array}$$

(5) $$\begin{array}{cc} \xi_{j} & =\left\{\begin{array}{cc} ∥y^{m}-y^{t}∥-\varepsilon & \mathrm{if}\;∥y^{m}-y^{t}∥>\varepsilon \\ 0 & \mathrm{otherwise} \end{array}\right. \end{array}$$

Here, ε and *C* are hyperparameters for each model. Deviations from the true value smaller than the threshold ε are neglected, and the regularization parameter *C* is a penalty for outliers, for which lower *C* values allow lower weights leading to a higher generalization. More details of the SVR method are given in Ref. [30].

The models are evaluated with the mean absolute error MAE and the Pearson correlation coefficient ρ,

(6) $$\begin{array}{ccc} \rho (y^{t},y^{m}) & = & \frac{\mathrm{cov}(y^{t},y^{m})}{\sigma_{y^{t}}\sigma_{y^{m}}}, \end{array}$$

(7) $$\begin{array}{ccc} \mathrm{MAE}(y^{t},y^{m}) & = & \frac{\sum_{i=1}^{N}∥y^{m}-y^{t}∥}{N_{\mathrm{samples}}}, \end{array}$$

using a 5-fold cross-validation. The hyperparameters for each of the ML models are optimized with respect to the MAE and ρ in a full grid search for various polynomial degrees *d*, regularization parameters *C* and insensitive error margins ε.

We refer to Ref. [1] for a validation of the predictions for fractional occupations, in which the same model architecture has been used on a system with a smaller configurational space, but the results should be transferable to our work. As a full validation for our case would need several million of further calculations, we instead use an uncertainty estimation to evaluate the robustness of our models. To estimate the uncertainty of the prediction at continuous values for site occupations, as opposed to the integer occupations present in the training data, we employed a bootstrap ensemble approach [31]. From the original training set of 2718 structures, we generated 50 bootstrap resamples by drawing *N* samples with replacement, where *N* equals the size of the training set. An independent SVR model with identical hyperparameters was trained on each resample. For a given input feature vector, the prediction uncertainty is then quantified as the standard deviation across the 50 model predictions. To systematically assess how this uncertainty depends on the degree of interpolation, we swept each impurity feature continuously from zero to its maximum site occupation (determined by the Wyckoff site multiplicity), while adjusting the corresponding base element occupation accordingly. For each sweep point, we computed the distance to the nearest endpoint, defined as $\delta =\min\left(x,x_{\mathrm{int}}-x\right)/x_{\mathrm{int}}$, where *x* is the continuous occupation value and $x_{\mathrm{int}}$ is the nearest considered integer value for that feature. This metric is zero at integer endpoint values where training data exist and 0.5 at the midpoint of the occupation range. The bootstrap uncertainty we report is calculated for δ = 0.25–0.5 to examine configurations far from the training datapoints in our descriptor space.

We additionally performed multi-feature sweeps over 500 random combinations of two, three, or four features each to account for multiple substitutions, still considering the constraint that the total occupation per site does not exceed its multiplicity. The distance metric was generalized as the maximum single-feature δ across all varied features, and 50 random points were evaluated per multi-feature combination.

## 3. Results and Discussion

### 3.1. Machine Learning Results

The optimized hyperparameters and the corresponding performance metrics are listed in Table 1. The correlation coefficients are close to one, and the MAEs are low compared to the range of possible values.

We estimate the uncertainty with the bootstrap approach outlined in Section 2.3. The resulting averaged standard deviations for the magnetic properties $M_{s}$ and $K_{1}$ listed in Table 1 are well below the reported MAE. This is an indicator of the model robustness for predictions in the continuous range of occupation values between the calculated integer values. We find the maximum standard deviations for substitutions of Nb (likely due to the low number of converged calculations in the training dataset), Mn, or Cr (likely due to reasons described in Section 3.2). We note that a possible systematic error of the SVR model would not be captured by this bootstrap uncertainty estimation.

**Figure 2:**
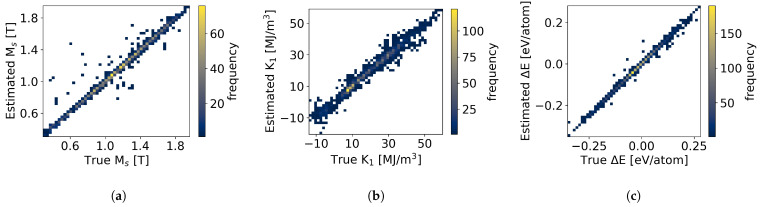
Cross validation of ML estimations for $M_{s}$, $K_{1}$ and ΔE plotted as 2D histogram. (**a**) $M_{s}$ estimation. (**b**) $K_{1}$ estimation. (**c**) ΔE estimation. The color indicates the frequency of true and estimated value pairs, where yellow bins include many configurations, and blue bins include few or only one configuration.

The data obtained with the three ML models for the three studied properties are plotted versus the test data in Figure 2 in 2D histograms. The three ML models perform equally well for the three properties.

**Figure 3:**
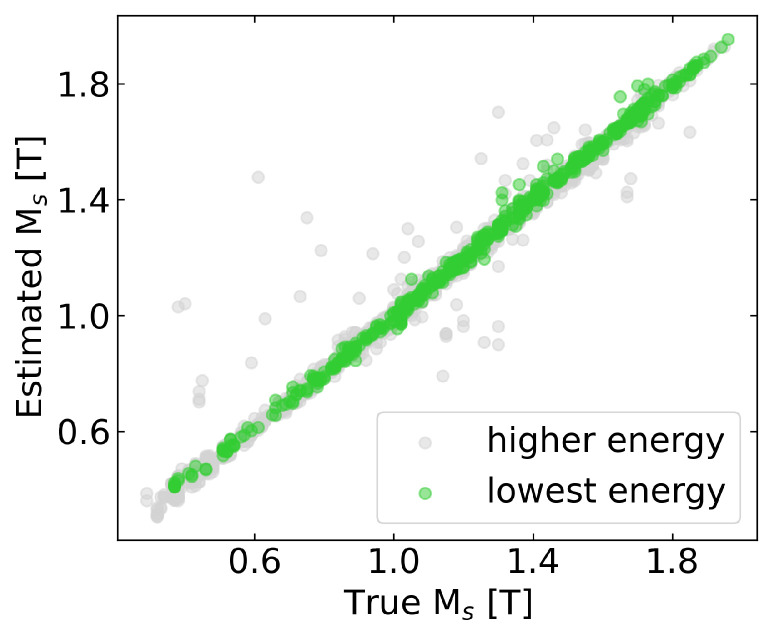
Cross validation graph for the estimations of $M_{s}$ for all configurations (grey) and only those configurations having the lowest energy for a fixed concentration of elements (green).

As seen in Figure 2, there are visible outliers, especially in the 2D histogram for $M_{s}$. Nevertheless, this is acceptable considering that we focus on low energy configurations, as explained in the following. Depending on the individual Wyckoff position occupied by an impurity element, the same chemical composition can have different phase formation energies $\Delta E_{f}$. The configuration with the lowest $\Delta E_{f}$ for a fixed concentration of elements is the most stable one. Focusing on the lowest-energy structures, we observe that their saturation magnetizations are estimated without severe outliers, as demonstrated in Figure 3. This is a good sign, because the low energy configurations should be the thermodynamically stable ones and, therefore, the interesting ones concerning the hard-magnetic properties.

The three ML models can be used to estimate the magnetic property changes for small changes in the concentration of elements, which would otherwise need explicit DFT calculations with large atomistic supercells. To describe a 0.1% change (relevant in recycling procedures), one would need 1000 atoms in a supercell to calculate the change in magnetic properties, while it is just a small adjustment in the input feature vector for the ML models.

### 3.2. Bimodal Distributions of $M_{s}$ for Cr and Mn

**Figure 4:**
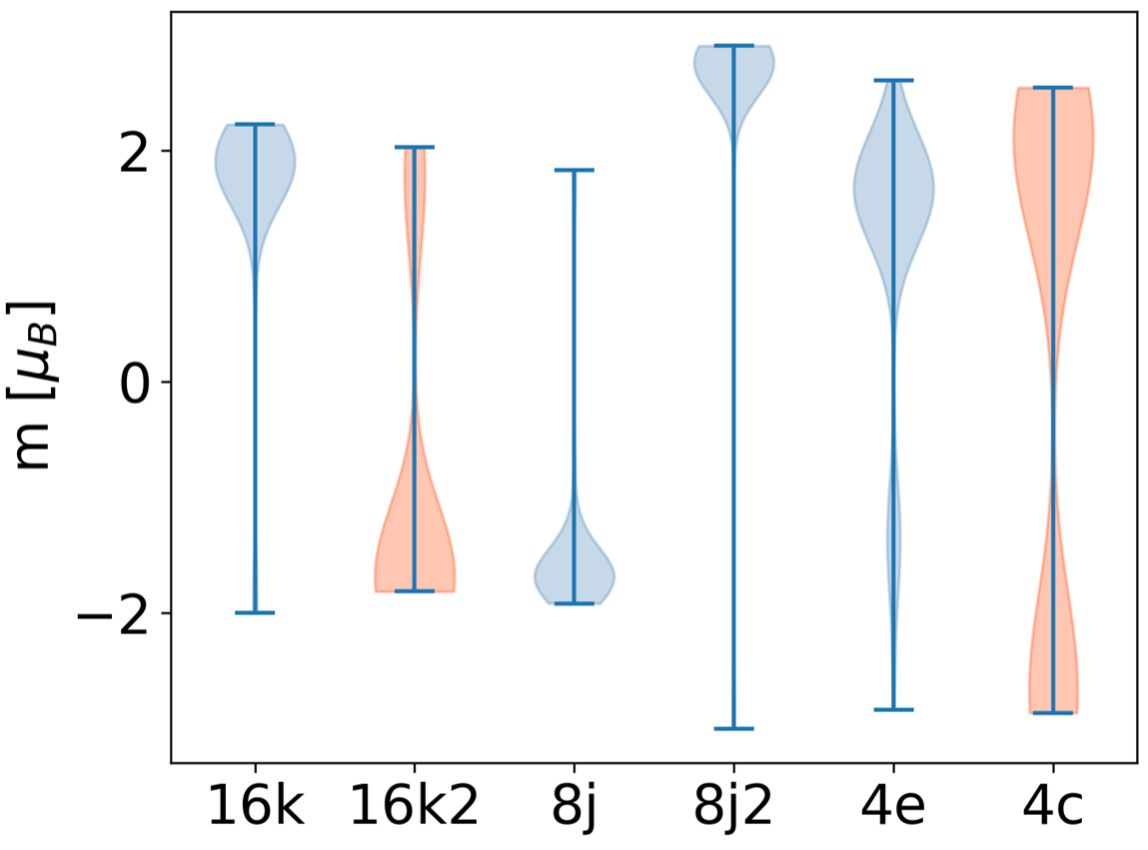
Distribution of atomic magnetic moments *m* of Mn atoms at different Wyckoff positions. All calculated configurations with Mn at the respective Wyckoff position are taken into account. Distributions highlighted in orange have significant amounts of positive and negative moments with a large discontinuity in between, making an estimation on these configurations less reliable if not handled differently.

The saturation magnetization depends on the orientation of the individual atomic magnetic moments in the permanent magnet. While all magnetic moments of ferromagnetic Fe point in the same direction, several elements that we substitute are antiferromagnetically coupled to their neighboring elements. If the substitutions have a high magnetic moment, the magnetization data contain positive or negative contributions of the individual features (element and Wyckoff position) with a large gap in between (see Figure 4). This bimodal distribution poses a difficulty for the learning process and leads to discrepancies between the DFT calculated and ML inferred values, especially for material compositions containing the elements Cr and Mn. We mitigate these discrepancies by introducing a prefactor for certain features, which decreases the MAE of the $M_{s}$ estimation by 19% (details are given in Appendix A). We note that this would happen automatically in other ML architectures [32] that learn weights for individual features rather than for similarities of entire feature vectors. However, the SVR models performed better than models such as neural networks and gradient boosted regression as quantified by the cross-validation metrics MAE and ρ, and with this method, the physical interpretation of our ML model is easier. Any tested chemical composition can be explained by its similarity to support vectors from the training dataset.

### 3.3. Discontinuities in $K_{1}$

A new capability of our ML approach is that it can describe the magnetic properties of compounds containing several rare-earth elements. The rare-earth elements naturally have the largest influence on the magnetic anisotropy of the permanent magnets, and therefore, the predictions of $M_{s}$ and $K_{1}$ can be improved, too, by introducing a prefactor for the features describing rare-earth elements (see Appendix A). The MAE for $K_{1}$ estimations decreases by 27% and particularly improves the outputs for Sm. According to the crystal-field anisotropy model of rare-earth atoms in intermetallic compounds, Sm favors a different magnetocrystalline anisotropy than Nd and the other rare-earth elements in our dataset. Magnetic 2-14-1 phases containing this element have negative $K_{1}$ values, which translates to an easy-plane magnetization, while the other rare-earth elements have highly positive $K_{1}$ values that stabilize an easy-axis magnetization. As a consequence, there also is a discontinuity between the positive and negative $K_{1}$ data, and this highlights how difficult it is to include rare-earth elements favoring an easy-plane magnetization. Elements that occupy a rare-earth Wyckoff position (4f and 4g) and have no 4f electrons like La do not contribute to $K_{1}$ because of the 4f anisotropy model included in our DFT calculation method [14], but they caused no problems for the ML models. The overall high sensitivity of $K_{1}$ on the elemental substitutions seen in the large range of values coincides with previous findings for other types of hard-magnetic intermetallic rare-earth transition-metal compounds that are useful for permanent magnets [27].

### 3.4. Current Limitations of the Model

We already showed that the three simple, fast and lean ML models accurately predict the three relevant properties, *K*_1_, *M*_s_ and Δ*E*_f_, for Nd_2_Fe_14_B-type magnets for a wide variety of substitutional elements. As stated in the previous subsections, discontinuities in $M_{s}$ and $K_{1}$ worsen the performance, but the difficulties can be handled. The unit cell volume is kept constant, which is problematic for larger elements like Zr, which we placed in rare-earth Wyckoff positions after comparing the stabilities of Nd- and Fe-substitution via the calculated $\Delta E_{f}$ values. It also appears reasonable, because it was experimentally confirmed that Zr substitutes Nd, not Fe, in ${\mathrm{NdFe}}_{12}$ [33]. We stress, however, that our calculated data are based on the hypothesis that all substituents are incorporated into the Nd_2_Fe_14_B-type crystal structure, which is not always given, as stated in Section 2.1.

The fixed size of the unit cell also has an influence on $\Delta E_{f}$ for different substitutions. This can result in elements being substituted at Wyckoff positions that are less stable and have a different influence on the magnetic properties than at the sites which are experimentally confirmed. An example of this is Ni, which should substitute on ${16k}_{1}$, ${8j}_{2}$ or 4e [34], but it is substituted on 4c in our lowest energy configuration with a higher increase of $M_{s}$ per substituted Ni atom. Note that this false hierarchy of energies has been found also in full-potential DFT calculations [35].

### 3.5. Multiple Substitutions

As the training dataset only includes calculations with one substitutional element in the otherwise pure ${\mathrm{Nd}}_{2}$ ${\mathrm{Fe}}_{14}$B crystal, ML predictions on crystals with more than one substitutional elements are more difficult. As an example, we compare combinations of Al, Ni and Co (hypothetically coming from incompletely separated AlNiCo magnets) at the 4c and 4e Wyckoff positions of Fe in Table 2.

The ML predictions are within a deviation of only 3% percent to our DFT calculated results in these magnets, even though these combinations are completely unknown to the ML model. This demonstrates the capability of this approach to significantly reduce the amount of necessary training data, because it can describe elemental combinations without being trained for this. Testing this with Mn, an element that made the predictions difficult, the largest deviation we got is 17%, if Mn and Ni are substituted.

The bootstrap uncertainty estimation for multiple substitutions (Table 3) rises monotonically with each additional substitution. We considered up to four features that are changed in composition here, and the uncertainty is still below the MAE of the models, which indicates a robustness of the model, but of course the prediction will be more uncertain for larger numbers of substitutions.

## 4. Conclusions

We presented a machine learning approach to predict magnetic properties successfully for a wide variety of elemental substitutions in ${\mathrm{Nd}}_{2}$ ${\mathrm{Fe}}_{14}$B. On one hand, this enables us to study the effect of small changes in impurity concentrations that would otherwise require expensive DFT calculations with large supercells. On the other hand, we can infer properties for combinations of different impurity elements without the need to calculate the intrinsic properties for this huge configurational space. Although the ML predicted values only describe intrinsic hard-magnetic properties of single-crystalline phases, it has been demonstrated that an incorporation of these properties can be used to infer extrinsic hard-magnetic properties of multi-crystalline microstructures [5,6].

Our work demonstrates the possibility of using relatively simple ML models and small datasets to accurately learn complicated relationships of chemical composition and uniaxial magnetization, even though the underlying coupling between magnetic moments at neighboring atoms differs significantly for the elements in the dataset. The ML models have MAE values close to zero and correlation coefficients close to one.

Difficulties in training intermetallic compounds containing transition-metal elements favoring antiferromagnetic coupling of magnetic moments (e.g., Cr and Mn) and rare-earth elements tending to magnetic easy-plane instead of easy-axis anisotropy (e.g., Sm) and means to overcome these difficulties in ML models were described and discussed.

## Figures and Tables

**Table 1:** Optimized hyperparameters for the three ML models. Listed are the degree *d* of the polynomial kernel, the regularization *C* and the loss insensitivity ε. Performance metrics are the correlation coefficient ρ and the MAE. The bootstrap uncertainty estimation for the continuous occupation values is given as the averaged and maximum standard deviation of predictions at the input values far from the calculated integer values in descriptor space.

Property	Hyperparameters			Cross Validation		Bootstrap Uncertainty	
**(Unit)**	** d **	** C **	** ε **	** ρ **	**MAE**	**Avg.**	**Max.**
$\mu_{0}M_{s}$ (T)	2	1	0.01	0.992	0.019	0.005	0.043
$K_{1}$ (MJ/$m^{3}$)	2	1000	0.5	0.986	1.5	0.3	1.4
$\Delta E_{f}$ (eV/atom)	2	10	0.001	0.998	0.003	0.002	0.038

**Table 2:** Saturation magnetization $\mu_{0}M_{s}$ calculated using TB-LMTO-ASA and predicted from the ML model is compared for combinations of substituted Al, Ni and Co at the 4c and 4e Wyckoff sites in the otherwise pure ${\mathrm{Nd}}_{2}$ ${\mathrm{Fe}}_{14}$B crystal.

Substitution		$\mu_{0}M_{s}$ (T)	
**4c**	**4e**	**ML Model**	**TB-LMTO-ASA**
Al	Co	1.63	1.68
Co	Al	1.68	1.63
Al	Ni	1.58	1.63
Ni	Al	1.62	1.57
Co	Ni	1.75	1.76
Ni	Co	1.75	1.75

**Table 3:** Bootstrap uncertainty estimation for $\mu_{0}M_{s}$ and $K_{1}$ when multiple elements are substituted.

No. of Substitutions	Avg. Bootstrap Uncertainty	
	**$\mu_{0}M_{s}$ (T)**	**$K_{1}$ (MJ/$m^{3}$)**
2	0.007	0.4
3	0.008	0.5
4	0.009	0.6

**Figure A1:**
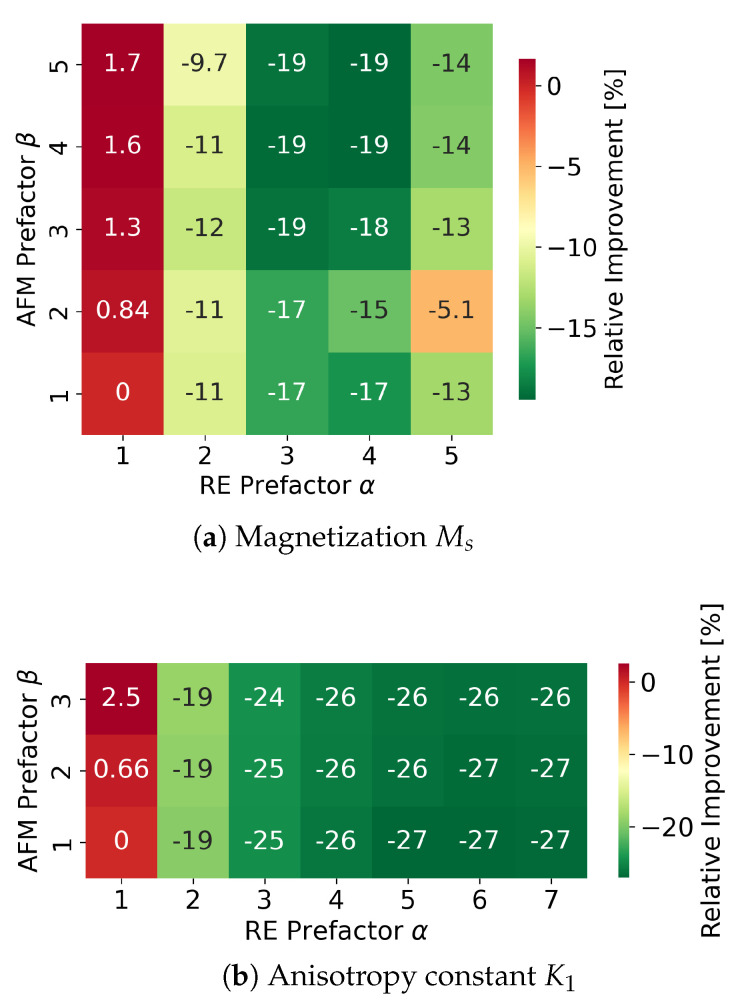
Heat maps showing the relative improvement of the MAE in the ML estimations of (**a**) the magnetization and (**b**) the anisotropy constant, when using different prefactors.

## Data Availability

The original contributions presented in this study are included in the article/Supplementary Materials. Further inquiries can be directed to the corresponding authors.

## Supplementary Materials

The following supporting information can be downloaded at https://www.mdpi.com/article/10.3390/ma19122643/s1. The file structures.extxyz contains all configurations in our dataset as readable crystal structures in extended xyz format. The file properties.csv contains information on which Wykoff positions are substituted in which configuration and the corresponding values of the three learned properties.

## Abbreviations

The following abbreviations are used in this manuscript:

ML	Machine Learning
DFT	Density Functional Theory
HTS	High Throughput Screening
TB-LMTO-ASA	Tight-Binding Linear-Muffin-Tin-Orbital Atomic-Sphere-Approximation
SVR	Support Vector Regression
MAE	Mean Average Error
RE	Rare Earth
AFM	Antiferromagnet

## Appendix A. Descriptor Optimization

As stated in Section 3.2 and Section 3.3, we introduce prefactors to certain input features to improve the MAE of the ML estimations for $M_{s}$ and $K_{1}$. One prefactor is for the rare-earth elements (RE prefactor α), and one prefactor is for selected positions of the antiferromagnetic elements Cr and Mn (AFM prefactor β).

For example, that means, that an original input feature vector

(A1) $$x=\langle \ldots ,n_{4g}^{\mathrm{Nd}}=4,\ldots ,n_{8j2}^{\mathrm{Cr}}=8,\ldots \rangle$$

with four atoms of Nd in the 4g site and eight atoms of Cr in the 8j2 site will be modified to read

(A2) $$x=\langle \ldots ,n_{4g}^{\mathrm{Nd}}=\alpha *4,\ldots ,n_{8j2}^{\mathrm{Cr}}=\beta *8,\ldots \rangle$$

This changes the way how an entry in the feature vector is weighted when applying the kernel function to determine the similarity to other support vectors.

Different values for the prefactors were evaluated, as shown in Figure A1. We performed the grid-search optimization of the hyperparameters outlined in Section 2.3 for each combination of prefactors for a fair comparison. Notably, the optimal *C* parameter in the ML model for the $M_{s}$ estimation decreases for α≥2 and β≥2 from 10 to 1 (we allowed the values: [−0.1, 1, 10, 100]). As a reminder, *C* is a penalty for outliers, and decreasing this parameter increases the generalizability of the model by allowing smaller weights. Apparently, higher weights are necessary for the support vectors including Cr and Mn without the prefactors, which results in an overfitting. This increased generalizability is an additional positive effect of this descriptor optimization, but the jump in the hyperparameter also results in an unintuitive increase in the MAE with an increase in β as seen e.g., for the (α=5,β=2) tuple in Figure A1a.
